# Community partnership approaches to safe sleep (CPASS) program evaluation

**DOI:** 10.1186/s40621-024-00528-y

**Published:** 2024-09-05

**Authors:** Gina S. Lowell, Jillian Sanford, Linda Radecki, Allison Hanes, Bonnie Kozial, Felicia Clark, Jennifer McCain, Asim Abbasi, Sevilay Dalabih, Benjamin D. Hoffman, Lois K. Lee

**Affiliations:** 1 Department of Pediatrics, Rush University Medical Center, 1645 W. Jackson Blvd, Suite 200, Chicago, IL 60612 USA; 2 Division of Pediatric Pulmonology and Sleep Medicine, Department of Pediatrics, Oregon Health and Science University, Portland, OR USA; 3 RadeckiResearch LLC, San Diego, CA USA; 4 American Academy of Pediatrics, Itasca, IL USA; 5 Department of Pediatrics, Division of Pediatric Emergency Medicine, University of Alabama at Birmingham, Birmingham, AL USA; 6 Department of Pediatrics, Division of Pediatric Emergency Medicine, University of Rochester Medical Center, Rochester, NY USA; 7 Arkansas Children’s Hospital, Pine Bluff Clinic, Pine Bluff, AR USA; 8 Department of Pediatrics, School of Medicine, and Doernbecher Tom Sargent Safety Center, Doernbecher Children’s Hospital, Oregon Health and Science University, Portland, OR USA; 9 Division of Emergency Medicine, Boston Children’s Hospital, Boston, MA USA

**Keywords:** SUID, SIDS, ASSB, Prevention, Safe sleep, Community partnerships

## Abstract

**Background:**

Sudden unexpected infant death (SUID) continues to be a leading cause of death in infants in the United States (US), with significant disparities by race and socio-economic status. Infant safe sleep behaviors are associated with decreasing SUID risk, but challenges remain for families to practice these routinely. The objective of this program was to implement and evaluate a novel approach for an infant safe sleep pilot program built upon partnerships between hospitals and community-based organizations (CBOs) serving pregnant and parenting families in at-risk communities.

**Methods:**

Community Partnership Approaches to Safe Sleep (CPASS) was a prospectively implemented infant safe sleep program. CPASS included children’s hospitals partnered with CBOs across five US cities: Portland, OR, Little Rock AR, Chicago, IL, Birmingham, AL, and Rochester, NY. The program consisted of (1) monthly learning community calls; (2) distribution of Safe Sleep Survival Kits; and (3) surveys of sites and families regarding program outcomes. Survey measures included (1) site participation in CPASS activities; (2) recipients’ use of Safe Sleep Kits; and (3) recipients’ safe sleep knowledge and behaviors.

**Results:**

CPASS learning community activities were consistently attended by at least two representatives (1 hospital-based, 1 CBO-based) from each site. Across the five sites, 1002 safe kits were distributed over 9 months, the majority (> 85%) to families with infants ≤ 1 month old. Among participating families, 45% reported no safe sleep location before receipt of the kit. Family adherence to nighttime safe sleep recommendations included: (1) no bedsharing (M 6.0, SD 1.8, range 0–7); (2) sleep on back (M 6.3, SD 1.7, range 0–7); and (3) sleep in a crib with no blankets/toys (M 6.0, SD 2.0, range 0–7). Site interviews described how participation in CPASS influenced safe sleep conversations and incorporated local data into counseling. Hospital-CBO relationships were strengthened with program participation.

**Conclusions:**

The CPASS pilot program provides a new, innovative model built on hospital-community partnerships for infant safe sleep promotion in SUID-impacted communities. CPASS reached families before their infant’s peak age risk for SUID and empowered families with knowledge and resources to practice infant safe sleep. Important lessons learned included improved ways to center and communicate with families.

**Supplementary Information:**

The online version contains supplementary material available at 10.1186/s40621-024-00528-y.

## Introduction

Sudden unexpected infant death (SUID) is the leading cause of death for infants one-month to one-year old, resulting in the death of approximately 3400 infants in the U.S. annually (Moon et al. 2022a). SUID is defined as the sudden and unexpected death of an infant less than one-year-old with no immediately obvious cause. The majority of SUID occur during sleep and are defined as infant deaths from: (1) accidental suffocation and strangulation in bed (ASSB), (2) ill-defined or unknown causes, or (3) sudden infant death syndrome (SIDS). ASSB is assigned when the infant’s death is caused by suffocation or asphyxia from obstruction of the nose and mouth, or compression of the neck or chest, by soft bedding, overlaying, wedging/entrapment, and/or strangulation. The latter two causes of death are assigned when there is no explanation, even after a full investigation.

The triple-risk model proposes that SIDS is likely to occur when three factors coincide: (1) the infant’s intrinsic vulnerability; (2) during a critical period of development; and 3) with an exogenous stressor (e.g. bed-sharing, soft bedding, prone sleeping) Filiano and Kinney 1994). Data from the Center for Disease Control and Prevention’s (CDC) SUID Case Registry showed that from 2011 to 2017, most (75%) SUID occurred among infants less than six-months-old. For SUID with complete case information, 98.5% occurred in an unsafe sleep environment (Parks et al. 2021).

In 1994 the American Academy of Pediatrics (AAP) collaborated with the National Institute of Child Health and Human Development (NICHD) and other stakeholders on the “Back-to-Sleep” public health campaign (Eunice Kennedy Shriver National Institute of Child Health and Human Development 2024) Afterwards infant supine sleep position use increased from ~ 15% in 1998 to ~ 72% in 2010 and the rate of SIDS (deaths per 1000 live births) decreased from ~ 1.4 in 1988 to ~ 0.5 in 2010 (Coverstone and Kemp 2019). Over the past two decades the combined SUID rate has plateaued and infant death rates attributed to unknown cause or ASSB have increased (Centers for Disease Control and Prevention 2024). Race and ethnic disparities persist with SUID rates over two times higher among non-Hispanic American Indian/Alaska Native (NH AI/AN) infants and approximately two times higher in non-Hispanic Black (NHB) infants, compared to non-Hispanic white (NHW) infants. In large US cities, these disparities are magnified with SUID rates for NHB infants three to 12 times that of NHW infants, and SUID rates for Hispanic infants consistently higher than NHW infants, a disparity not seen in the national data (Boyer et al. 2022). These disparities are rooted in inequitable access to trusted information and resources (Menon et al. 2023), and are further challenged by low awareness of SUID as a leading threat to the lives of infants (Quinlan et al. 2018).

The AAP’s most recent guidelines continue to reinforce promoting safe sleep practices anchored in placing infants to sleep on their backs, on a firm, non-inclined sleep surface, without soft bedding or other items in their own sleep space (Moon et al. 2022b) While public health campaigns, newborn nurseries, or primary care offices are common avenues where safe sleep recommendations are delivered, there is evidence that community-driven efforts, such as community agency baby showers or peer counseling, may be more effective (Menon et al. 2023). To this end, the AAP launched the Community Partnership Approaches to Safe Sleep (CPASS) Program. The objective of CPASS was to develop and implement a novel approach by establishing authentic partnerships between injury prevention experts at children’s hospitals with community experts at community-based organizations (CBOs) serving communities with disproportionately high SUID rates to deliver infant safe sleep education and resources.

## Methods

This was a one-year prospective pilot program from December 2021–December 2022 with a formative evaluation of five children’s hospital-CBO partnerships for an infant safe sleep education and product distribution intervention. From a call for applications among Injury Free Coalition for Kids hospital sites, five children’s hospitals and their CBO partners were selected to participate in this study. Participants from each hospital included the Injury Free Coalition for Kids site Principal Investigator (PI), CPASS site PI, and injury prevention program staff; CBO participants included center directors, program specialists, parent and peer educators, and/or community health workers. The sites were chosen for their demonstrated need based on local SUID data from their community, and hospital and CBO service to families from SUID-impacted populations. Funding for the program was provided by Amazon to the AAP, who disbursed funds to each of the CPASS sites to participate in the program. Amazon had no role in the product selection. This study was approved by the institutional review board of the AAP.

### CPASS program implementation

**Fig. 1:**
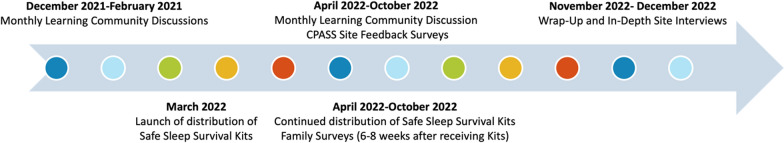
CPASS learning community, safe sleep survival kit distribution, and data collection timeline

The CPASS program model included three components: (1) implementation of the CPASS learning community; (2) distribution of the Safe Sleep Survival Kits; and (3) data collection from CPASS site and family participants. (Fig. 1) To implement the CPASS learning community, AAP program staff held monthly calls with the five CPASS sites for 11 months. The goals of the CPASS learning community were to share knowledge, best practices, and discuss challenges and successes associated with program implementation grounded in providing timely and equitable access to safe sleep education and resources.

Pregnant and postpartum individuals received Safe Sleep Kits and participated in site-specific educational activities between April and August 2022. Families were eligible if the parenting individual was in their third trimester, already had a newborn, or if they had infants two to 12 months old and a need for a Safe Sleep Kit. Each site developed their own outreach and engagement approaches for families, capitalizing on their knowledge of and relationships with their community.

**Fig. 2:**
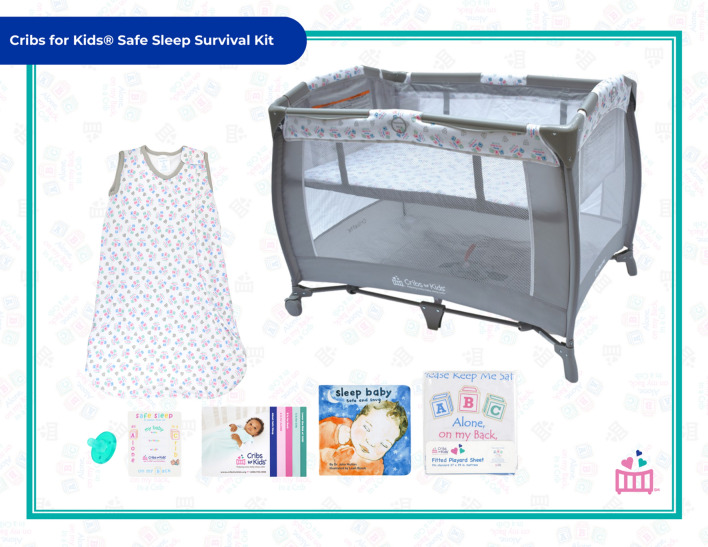
Cribs for kids safe sleep survival kit

Safe Sleep Survival Kits were obtained through Cribs for Kids (Fig. 2) and were purchased directly by the CBO and/or children’s hospital through an online portal for the CPASS program. Each hospital-CBO site received funds to purchase 200 kits for a total of 1000 kits across the entire CPASS program. While Cribs for Kids educational materials were initially translated only into Spanish, CBOs recognized a need for additional language translations. By CPASS conclusion, Cribs for Kids educational materials were translated into five additional languages (Amharic, French, Russian, Swahili, and Tigrinya). Safe Sleep Kits were either mailed directly to program participants’ homes or distributed by the hospital or CBO by another method (e.g. community baby shower). Distribution methods were at the hospital and CBO discretion based on their knowledge of community needs.

### CPASS program evaluation

CPASS used a formative evaluation approach to assess program implementation and to examine the early program impacts of site partnerships and Safe Sleep Kit distribution. The evaluation plan was anchored by the expected outcomes of the program activities based on the three aims in the CPASS logic model (Additional file 1): (1) fidelity to the CPASS program model; (2) safer sleep for babies through community partnerships; and (3) enhancing and sustaining the future of CPASS. Questions regarding fidelity to the CPASS model focused on the learning community model implementation and site participation in learning community activities. For babies’ safer sleep, the questions focused on the successful distribution of the Safe Sleep Kits, survey responses by participating families, and any changes in the relationships between site partners due to CPASS. To evaluate the future of CPASS, questions focused on lessons learned from participating sites and families to inform revision of the learning model and program and to improve AAP safe sleep-related outreach.

The evaluation included four primary data collection components: (1) project documentation; (2) post-learning community call participant online feedback surveys (Additional file 2); (3) Safe Sleep Kit recipient family surveys, available in English and Spanish (Additional file 3); and (4) in-depth site interviews at CPASS conclusion. Descriptive frequencies were calculated for quantitative data. Project documentation included attendance records at monthly learning community calls and notes from these calls. For the learning community surveys, responses were compiled monthly and shared with program staff and leaders. To allow families time to use the Safe Sleep Kits, recipients were surveyed six to eight weeks after distribution using a survey link disseminated either by email or as a text message to the participant, based on their stated preference. A reminder was sent one week later to optimize survey response. Each hospital-CBO site was responsible for tracking kit distribution, survey dissemination, survey response, and one week follow up. Families who received their Safe Sleep Kit after September 1, 2022 did not complete surveys as that was beyond the pre-determined end date for data collection and analysis. Semi-structured interviews with CPASS hospital and CBO representatives on a voluntary basis, were conducted by the CPASS program evaluator (L.R.). After informed consent was verbally obtained, interviews were recorded and transcribed. These transcripts were analyzed, both individually and in aggregate, for key themes and content.

## Results

Five children’s hospitals and their CBO partners participated in the CPASS program across five US cities: (1) Portland, OR—Doernbecher Children’s Hospital & Healthy Birth Initiatives; (2) Little Rock AR—Arkansas Children’s Hospital & Turning Point Youth Center; (3) Chicago, IL—Rush University Children’s Hospital & Family Focus; (4) Birmingham, AL—Children’s of Alabama & Birmingham Healthy Start Plus; and (5) Rochester, NY—Golisano Children’s Hospital & Baby Safe Sleep Coalition. There was strong site participation in the CPASS learning community activities with at least two representatives (one hospital-based, one CBO-based) from each site attending every call. CPASS site participants reported value in their CPASS experience: 96% strongly agreed/agreed that they learned something new from the calls and that the calls provided infant safe sleep-related information applicable to their work with families. In addition, 87% of respondents strongly agreed/agreed that the calls provided actionable strategies to contribute to infant safe sleep work in the community.

**Fig. 3:**
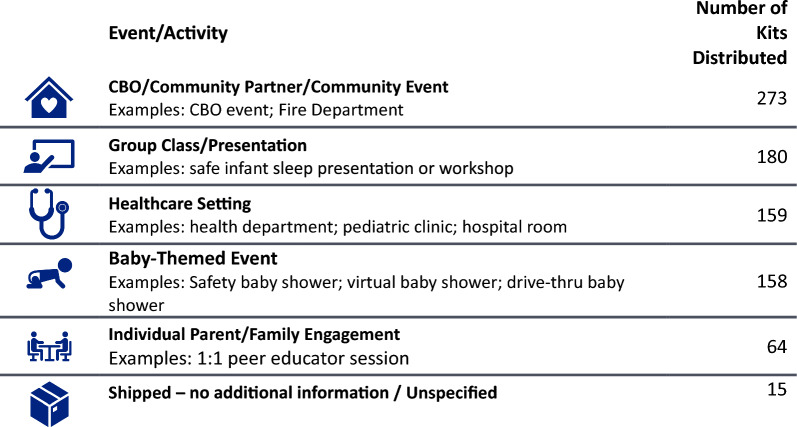
Safe sleep kit distribution methods

CPASS sites reached families at community events, group classes, healthcare settings, baby-themed events, and through individual engagement. (Fig. 3) Across the five sites, 1002 Safe Sleep Kits were distributed. Over 85% of Safe Sleep Kits were distributed to families who were pregnant or postpartum with infants less than one-month-old.

**Fig. 4:**
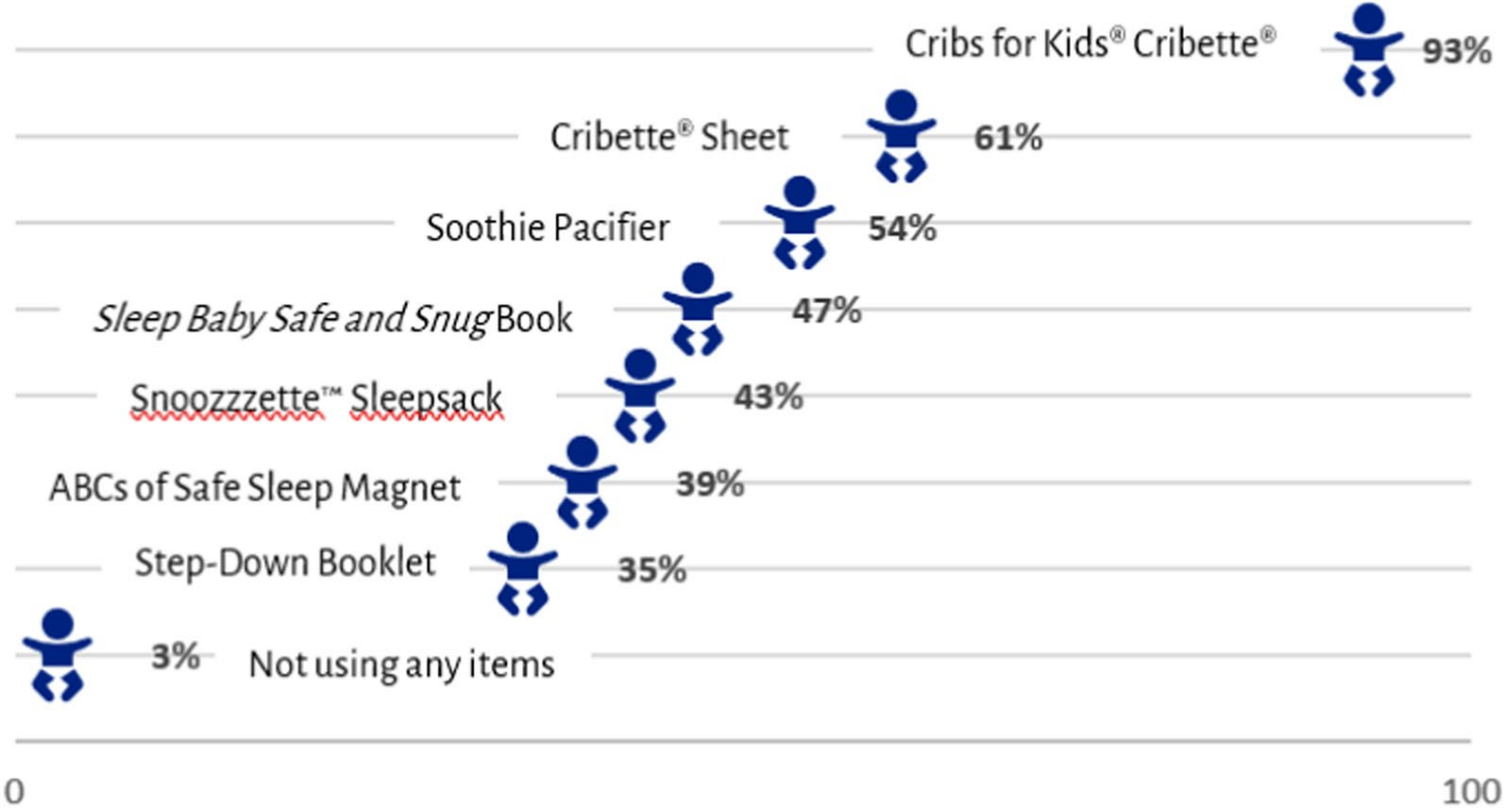
Use of safe sleep kit components by recipients

Among participating families, 168 (16.8%) completed the Family Survey. Among respondents, 75 (45%) reported having no safe sleep location before receipt of the Safe Sleep Kit. Respondents reported learning new infant safe sleep knowledge regarding no bedsharing (30%); sleeping on back (27%); and no items in the sleep environment (25%) (Table 1). Family adherence (in nights per week) to nighttime safe sleep recommendations and in daytime naps were similar in terms of mean nights or days (Table 2). There was variable uptake in the use of the Safe Sleep Kit items, and 93% of families reported using their cribette (Fig. 4).

**Table 1:** Safe sleep kit recipient knowledge survey

Survey questions	Responses (N = 168)	
Until 1 year, babies should sleep in their own crib/bassinet, and not bed-share with an adult.	Knew prior to Sleep Kit receipt: Learned upon receipt: Did not know:	68% 30% 1%
Until 1 year, babies should sleep on their backs.	Knew prior to Sleep Kit receipt: Learned upon receipt: Did not know:	68% 27% 5%
Until 1 year, babies should sleep on a flat surface, with a firm mattress and fitted sheet, with no blankets or toys.	Knew prior to Sleep Kit receipt: Learned upon receipt: Did not know:	72% 25% 3%

**Table 2:** Safe Sleep Kit Recipient Safe Sleep Practice Survey (from 168 recipients surveyed)

Sleep behavior/environment	Nighttime sleeping	Daytime napping
Sleeps in crib/bassinet, no bed-sharing	Mean nights = 6.0 SD = 1.8 Range = 0–7	Mean days = 6.0 SD = 1.8 Range = 0–7
Starts out placed on back	Mean nights = 6.3 SD = 1.7 Range = 0–7	Mean days = 6.0 SD = 1.9 Range = 0–7
Sleeps in crib, firm mattress, fitted sheet, no blankets/toys	Mean nights = 6.0 SD = 2.0 Range = 0–7	Mean days = 5.9 SD = 2.0 Range = 0–7

All five sites completed an in-depth interview. Qualitative review of in-depth interviews demonstrated the following accomplishments across sites:

- Deployment of trusted and passionate professionals who are in the community for delivering safe sleep messaging and resources.
- Hiring peer-educators to provide safe sleep education and support.
- Identifying and sharing local SUID data to raise family and community awareness; sites reported that communicating local SUID data was more impactful than conveying national or individual state data.
- Expanding partnerships and building relationships with other CBOs and community service providers.
- Taking an intergenerational approach to infant safe sleep messaging and education by offering community workshops, presentations, and educational sessions to caregivers across the age continuum from youth to young families to seniors.

CPASS participation influenced key changes in the ways that partners provide safe sleep education and counseling. As one site participant noted, their commitment to and engagement with community families was about “…so much more than handing off a crib.” Sites described moving from building awareness to enabling action and celebrating and building upon family safe sleep knowledge. Approaches including reframing infant safe sleep education as injury prevention with greater incorporation of concepts like ‘preventing suffocation’ were found to be useful with families.

CPASS participation strengthened hospital-CBO relationships, with sites reporting deeper, more trusting relationships at the program’s conclusion. Four of the five sites had established plans to continue collaborating on issues of SUID prevention and infant safe sleep promotion. All partner sites voiced concern regarding the inability of their limited funding to match their community need for safe sleep products. Partner sites perceived that the Safe Sleep Kits were the “key” that opened the door to greater relationship building with families and other organizations: “CPASS helped us to take things to a whole ‘nother level… We talk to [families] about sleep education and alternative sleeping methods other than co-sleeping. It was one thing talking about that, but it was another thing providing the equipment for them to be able to do so.”

## Discussion

The CPASS program successfully supported five hospital-CBO partnerships to reach over 1000 families from SUID-impacted communities with Safe Sleep Kits and education. CPASS sites provided families with these resources before their infant’s peak risk for SUID, with over 85% of families receiving Safe Sleep Kits before their infants were one-month-old. Safe Sleep Kit contents were highly utilized and cribette use was reported by 93% of recipients. Broad consensus among CPASS site participants indicated that beyond the availability of sleep kits, the monthly learning community calls were the most positive aspect of the program model. Site representatives affirmed the call environment fostered sharing and openness about safe sleep challenges and successes and inspired new and innovative ideas as a result of learning from other sites and program leaders.

Prevention recommendations to address evidence-based, modifiable risk factors for sleep-related deaths exist, but infant safe sleep guidance does not reach all parents and caregivers equally and in ways that resonate and foster attitudinal and behavioral changes (Moon et al. 2022a; Menon et al. 2023). Familial practices and traditions along with the demands of caring for newborns who wake frequently throughout the night are just two additional factors that contribute to bed-sharing, a risk factor for both suffocation-related and unexplained SUID (Parks et al. 2023). Children’s hospitals typically incorporate safe sleep education in postpartum or newborn care settings. However, they may not be knowledgeable of or intentionally partner with community agencies who serve pregnant and parenting families. The CPASS CBO partners are agencies supporting pregnant and parenting individuals by providing a range of services such as home visiting and doula services; parenting groups; family engagement; parent–child relationship support; community advocacy; safe sleep and resource supports. Such programs typically have funding from federal, state, grant and private funding sources. Their aims are grounded in improving disparities in maternal-child health outcomes through efforts that center and support families’ social, economic, educational and resource needs. The experience and wisdom of these community-based and community-trusted agencies expand opportunities for safe sleep promotion at a range of outreach events where safe sleep education can be provided. These events include community resource fairs; community baby showers; faith-based events; parenting or father’s group classes; and individual engagement. Such events and activities are geared towards meeting families where they are, both geographically (in neighborhoods where families live and at events families attend) and individually—understanding and considering how the real circumstances of families’ lives impact their ability to access and incorporate knowledge and resources to support and protect their health.

The CPASS Learning Community calls allowed for information sharing between the grantor and the sites, as well as for information exchange between sites. A highly useful aspect of the calls was learning how different sites in different cities were approaching outreach and family engagement, as well as hearing about CPASS site challenges and successes. Both hospital and CBO leads participated in every call from all five sites, reflecting the perceived high value of participation in the learning community.

An important focus of discussions was to reframe prevention messaging to emphasize how safe sleep practices ‘prevent suffocation’, rather than only reviewing that safe sleep practices prevent SIDS. Parents and caregivers may more easily picture how soft bedding, bedsharing and inclined surfaces can lead to suffocation. Understanding how these factors lead to SIDS—both because it is unclear how something with an unknown cause could be caused by certain risk factors, and because SIDS has been felt to be a tragedy that is random and outside of a parent’s control, may be difficult (Moon et al. 2010). Parks et al.’s updated case control study using PRAMS and SUID Case Registry data identified strongly overlapping risk factors for both suffocation-related SUID and unexplained SUID, implying that preventing suffocation-related infant deaths will likely also prevent unexplained SUID (Parks et al. 2023). Other shared best practices included approaches that centered families’ knowledge, beliefs and practices to start conversations that could build upon these values. Such conversations shift the focus from safe sleep education to safe sleep guidance—allowing for greater input and problem-solving from families in creating a more sustainable safe sleep environment for their infants.

Limitations of the CPASS program evaluation includes limited data capture of the actual number of families reached with safe sleep guidance. While 1002 families received the Safe Sleep Kits, outreach events including community events, group classes and baby showers would have included many more families who received safe sleep education but who did not receive a Safe Sleep Kit. Also, while this variability in the delivery of the intervention could limit a standardized evaluation, it also allowed for evaluation of community-participatory approaches to supporting families in practicing safe sleep. Further limitations include low completion of Family Survey responses, with just 16% of recipients completing the survey. The Family Survey was only offered in English and Spanish, excluding participation of other language recipients. Despite these limitations, CPASS provides an important new model for building strategic partnerships at the community level and empowering families with the education and resources needed to reduce infant mortality due to sleep-related deaths.

## Conclusions

This novel partnering of children’s hospitals with community-based organizations to provide safe sleep guidance and resources in places where families live and gather helped to increase access to trusted information and resources for families from SUID-impacted communities. CPASS reached families before their infant’s peak age risk for SUID and surfaced improved ways to reframe conversations and center families. In addition, this type of collaboration strengthened hospital-community relationships. Commitment to ongoing partnerships may sustain successful community outreach to promote safe sleep, but limited resources remain as a substantial challenge to providing tangible tools to support safer sleep and program sustainability. This model could also be applied to other pediatric injury prevention efforts to address health disparities and improve health outcomes for all children.

## Supplementary Information

Additional file 1. CPASS Logic Model

Additional file 2. CPASS Learning Community Feedback Survey

Additional file 3. CPASS Infant Safe Sleep Parent Family Survey

## Data Availability

The data that support the findings of this study are available from the American Academy of Pediatrics (AAP) and are available from the authors upon reasonable request and with permission of the AAP.

## Abbreviations

AAP: American academy of pediatrics
ASSB: Accidental suffocation and strangulation in bed
CPASS: Community partnership approaches to safe sleep
SIDS: Sudden infant death syndrome
SUID: Sudden unexpected infant death
NH AI/AN: Non-Hispanic American Indian/Alaska native
NHB: Non-Hispanic black
NHW: Non-Hispanic white
