# Supplementary material for: Community partnership approaches to safe sleep (CPASS) program evaluation
Source: Inj Epidemiol. 2024 Sep 5;11(Suppl 1):45. doi: 10.1186/s40621-024-00528-y (PMC11375816; doi:10.1186/s40621-024-00528-y)
Supplement: Supplementary file 1 — Additional file 1. CPASS Logic Model [file 40621_2024_528_MOESM1_ESM.pdf]

Logic Model – Community Partnerships Approaches for Safe Sleep (CPASS) Pilot

Program Goal: Bring together the expertise of children's hospitals, community-based organizations (CBOs), and child health experts to equip expectant parents and families of infants with the information and resources needed to implement and maintain safe sleep practices.

Specific Aims: 1) By November 2022, at least one member from each CPASS partner sites will participate in monthly learning community calls (≤13) for cross-site sharing of successes, challenges, materials, and resources to promote culturally-relevant infant safe sleep messaging and education; 2) By November 2022, five CPASS partner sites will provide individual- and community-based safe sleep education, trainings, and activities and distribute 200 Safe Sleep Survival Kits (1,000 kits total) to improve parent/caregiver knowledge and implementation of safe sleep practices; 3) By January 2023, AAP will apply pilot lessons learned to refine the CPASS community-based program model that engages childrens hospitals and CBOs in partnership to promote knowledge and consistent implementation of infant sleep practices with expectant parents and families of infants.

Inputs

CPASS Partner Sites*
- Alabama
- Arkansas
- Illinois
- New York
- Oregon

AAP Staff Support

Existing Expertise and Leadership
-AAP policy statements and clinical guidelines to promote safe sleep practices and environments
- Injury prevention and infant sleep expertise (individuals, children's hospitals, community-based organizations (CBOs), national organizations)

Existing Evidence Base
- Scientific literature documenting safer sleeping practices and environments
- Scientific literature documenting continued inequities in SUIDs

Funding
Amazon

Key Activities

CPASS Learning Community Calls
Monthly virtual convenings with topical safe sleep focus and opportunity for group sharing/discussion of successes, challenges, materials and resources

CPASS Partner Site Safe Sleep Community Training(s) and Activities

CPASS Partner Site Safe Sleep Survival Kit Distribution

CPASS Leadership Planning Calls
- Planning calls between AAP team and safe sleep/injury prevention leaders to develop project goals, content, and activities

Outputs

Hospital-CBO relationships

Peer-support network across CPASS partner sites

Culturally sensitive and appropriate safe sleep *materials* and *resources*

Culturally sensitive and community-focused safe sleep *trainings* and *activities*

Distribution of 1,000 Cribs for Kids Safe Sleep Survival Kits to expecting parents and families of infants (200 kits/CPASS partner site)

CPASS model program for replication and future expansion, scale

Short-Term Outcomes (Immediate to 1 month)

CPASS Partner Sites
Hospitals: Clinical expertise supports the safe sleep work of CBOs; hospitals recognize the key role that CBOs play in direct outreach to families
CBOs: Families identify CBO as source of safe sleep information and resources
Partnership: Shared information, resources, and relationship building contributes to increased knowledge and efficacy in developing and providing culturally sensitive and relevant education, trainings and activities to promote infant sleep to expectant parents, families of infants, and community members

Expectant Parents | Parents and Caregivers of Infants
- Acceptance of Safe Sleep Survival Kit
- Increased knowledge regarding safe sleep practices

AAP
Identification of learnings, successes and challenges from individual CPASS partner sites and the CPASS program, collectively

Intermediate Outcomes (3-6 months)

CPASS Partner Sites
Hospitals: Identify opportunities for greater community outreach to families through CBOs
CBOs: Families trust CBO as a source for safe sleep guidance and support
Partnership: Increased mechanisms to create and provide infant safe sleep education, trainings, activities and resources that meet the unique needs of families and community members and contribute to increased adoption of safe sleep practices

Expectant Parents | Parents and Caregivers of Infants
Adoption and consistent implementation of infant safe sleep practices

AAP
Increased understanding and promotion of community models to promote infant safe sleep to families and community members

Long-Term Outcomes (1-2 years)

CPASS Partner Sites
Hospitals: Recognized advocate for safe sleep and injury prevention by families
CBOs: Expanded relationships with local CBOs to support safe sleep/injury prevention
Partnership: Identify other opportunities to collaboratively support injury prevention & child health in community

Expectant Parents | Parents and Caregivers of Infants
- Implement safe sleep practices until child reaches appropriate age
- Implement safe sleep practices w/ younger siblings
- Share infant safe sleep messaging with others

AAP
Replication and scale of CPASS model for safe sleep and other injury prevention topics

Impact (aspirational)

Improved SUIDs rates in communities implementing the CPASS model

Fewer parents, family members, healthcare professionals, family-serving professionals, and community members grieve the preventable death of an infant

Improved child health and well-being

Improved materials and resources that offer culturally sensitive and relevant messaging for families and communities regarding infant safe sleep practices

Community, state and national organizations have the resources and infrastructure needed to promote infant safe sleep and support families in adopting infant safe sleep practices in homes and other settings (eg, childcare, shelters, hospitals)

*** CPASS Partner Sites**
- Arkansas Children's Hospital & Turning Point Youth Center
- Children's of Alabama & Birmingham Healthy Start Plus
- Doernbecher Children's Hospital & Healthy Birth Initiatives
- Golisano Children's Hospital & Baby Safe Sleep Coalition
- Rush University Children's Hospital & Family Focus
