# Supplementary material for: Community partnership approaches to safe sleep (CPASS) program evaluation
Source: Inj Epidemiol. 2024 Sep 5;11(Suppl 1):45. doi: 10.1186/s40621-024-00528-y (PMC11375816; doi:10.1186/s40621-024-00528-y)
Supplement: Supplementary file 2 — Additional file 2. CPASS Learning Community Feedback Survey [file 40621_2024_528_MOESM2_ESM.pdf]

CPASS Learning Community Feedback Survey

Thank you for joining the **October** CPASS Learning Community call! To help us understand your experience and improve future activities, please complete the following questions.

* 1. Please rate your level of agreement with the following statements:

	Strongly disagree	Disagree	Neither disagree nor agree	Agree	Strongly agree
I learned something new from this CPASS Learning Community call	○	○	○	○	○
This CPASS Learning Community call provided information that I will apply in my safe sleep work with families.	○	○	○	○	○
This CPASS Learning Community call provided actionable strategies that will contribute to safe sleep work in our community.	○	○	○	○	○

* 2. On a 1-7 scale where 1=low return on investment (ROI) and 7=high ROI, what was the return on your investment of time/effort for participating in this CPASS Learning Community call?

1 - **Low** return on investment	2	3	4	5	6	7 - **High** return on investment
○	○	○	○	○	○	○

* 3. Please share the **one** most important thing that you took away from today's call.

4. Please share any other thoughts about CPASS here.

* 5. What is your CPASS role?

Thank you for the time you have taken to share your feedback throughout CPASS!
We appreciate you and all that you do for children and families in your community!
