# Supplementary material for: Community partnership approaches to safe sleep (CPASS) program evaluation
Source: Inj Epidemiol. 2024 Sep 5;11(Suppl 1):45. doi: 10.1186/s40621-024-00528-y (PMC11375816; doi:10.1186/s40621-024-00528-y)
Supplement: Supplementary file 3 — Additional file 3. CPASS Infant Safe Sleep Parent Family Survey [file 40621_2024_528_MOESM3_ESM.pdf]

Infant Safe Sleep Parent / Family Survey

Hello and congratulations on the birth of a new baby in your family!

As a parent or other caregiver, we know that you want the very best for this child. The American Academy of Pediatrics and folks in your community are working together to help babies sleep safer.

Please answer the following questions about your experience with the Cribs for Kids® safe sleep kit. Completing this survey is voluntary. The information from this survey is confidential which means that we will not connect your name to information that you share. Answer each question to the best of your ability.

Your answers to this survey will be combined with information from other families to understand more about this safe sleep project. **Please do not share any personal information such as names, street address, telephone numbers, e-mail addresses, etc in your answers.**

Thank you!

* 1. Did you receive a Cribs for Kids® safe sleep kit?

◯ Yes

◯ No

Infant Safe Sleep Parent / Family Survey

* 2. Did you receive the safe sleep kit in English, Spanish, or another language? [please check all that apply]

☐ English

☐ Spanish

☐ Another language (describe here)

* 3. In what month did you receive your Cribs for Kids® safe sleep kit? If you can't remember for sure, please use your best guess.

* 4. How old is the baby now?

Infant Safe Sleep Parent / Family Survey

* 5. Which Cribs for Kids® safe sleep kit item(s) are you currently using? [please check all that apply]

☐ 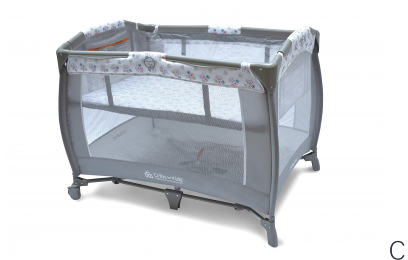

Cribs for Kids® Cribette®

☐ 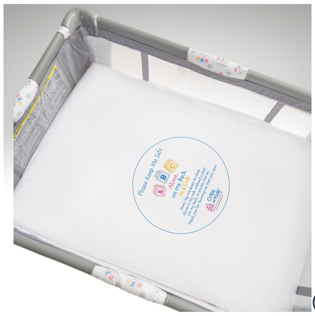

Cribette® Sheet

☐ 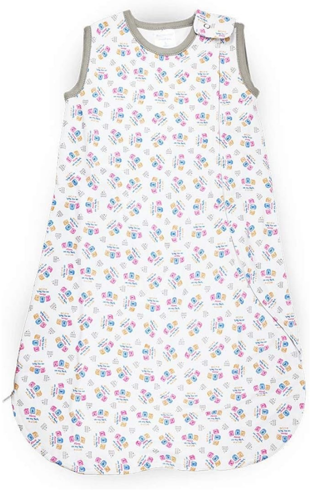

Snoozzzette™ SleepSack or Swaddlette Baby Swaddle Wrap

- [ ] 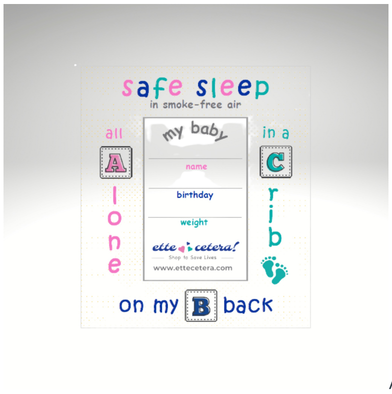

ABCs of Safe Sleep Photo Magnet

- [ ] 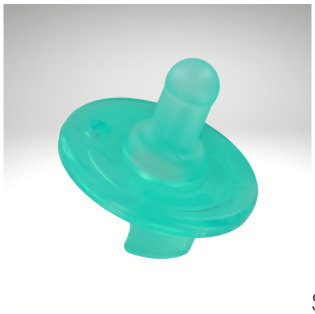
Soothie Pacifier

- [ ] 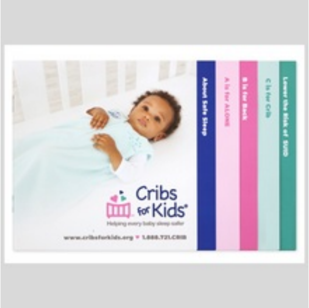

Step-Down Booklet

- [ ] 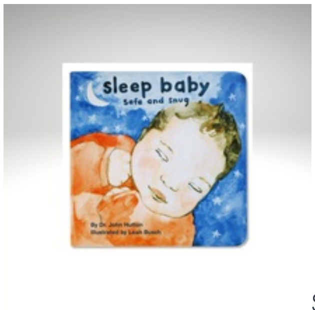

Sleep Baby Safe and Snug Children's Book

- [ ] Not using any safe sleep kit items

6. If you are not using the safe sleep kit items, please tell us why.

[ ]

* 7. Did the baby have a safe place to sleep before you received the Cribs for Kids® Cribette?

◯ Yes

◯ No

* 8. This question is about nighttime sleeping. How many nights a week does the baby:

	Number of nights
Sleep in his/her crib/cribette/bassinet and not share a bed with an adult or other child?	
Start out placed on the back, not tummy or side?	
Sleep in a crib/cribette or other safe-sleeping product with a firm mattress, fitted sheet and no blankets, toys, or stuffed animals?	

* 9. This question is about daytime naps. How many days a week does the baby:

	Number of days
Nap in his/her crib/cribette/bassinet and not share a bed with an adult or other child?	
Start out placed on the back, not tummy or side?	
Nap in a crib/cribette or other safe-sleeping product with a firm mattress, fitted sheet and no blankets, toys, or stuffed animals?	

10. What is the hardest part about putting the baby to sleep safely?

11. What questions do you have about putting the baby to sleep safely?

Infant Safe Sleep Parent / Family Survey

* 12. Did you know?

	I did not know this	I learned this when I received the safe sleep kit	I knew this before I received the safe sleep kit
Until the age of 1 year, babies should sleep in their own crib, cribette, or bassinet, and not share a bed with an adult or other child.	◯	◯	◯
Until the age of 1 year, babies should sleep on their backs, not on their tummies or sides.	◯	◯	◯
Until the age of 1 year, babies should sleep in a flat crib/cribette or other safe-sleeping product with a firm mattress, fitted sheet and no blankets, toys, or stuffed animals.	◯	◯	◯

* 13. What is your relationship to the baby?

◯ Mother

◯ Father

◯ Grandparent

◯ Aunt, uncle, cousin or other extended family member

◯ Foster parent

◯ Another relationship (describe here)

Thank you for your time and interest in infant safe sleep!

If you would like to talk with someone from [**CBO or Hospital**] about safe sleep or have more questions about helping your baby sleep safely, please contact click [**here**] for your local Health Educator's contact information.
